# Editorial: Nutrition, mental health, and long-term prognosis among cancer survivors

**DOI:** 10.3389/fnut.2024.1431466

**Published:** 2024-05-29

**Authors:** Ji-Bin Li

**Affiliations:** ^1^Department of Clinical Research, Sun Yat-sen University Cancer Center, Guangzhou, China; ^2^State Key Laboratory of Oncology in South China, Collaborative Innovation Center for Cancer Medicine, Guangzhou, China

**Keywords:** nutrition, nutritional support, mental health, prognosis, cancer survivors

In recent decades, the prognosis of patients with cancer has improved considerably due to advances in early screening, diagnosis, and therapeutic strategies, leading to a substantial increase in the number of cancer survivors (1). In 2018, it is estimated that there were around 43.8 million cancer survivors worldwide (2). However, life after a cancer diagnosis remains a significant challenge for cancer survivorship (3). Malnutrition and psychological issues (e.g., depression, anxiety, insomnia, and fear of recurrence) are two main challenges that are overlooked in clinical practice among this population. Poor nutritional status and mental health could significantly reduce the treatment efficacy and overall survival (4, 5), potentially leading to other complications (6). Furthermore, malnutrition is also associated with mental disorders (7). Despite this, the interrelationships between nutrition, mental health, and their impact on outcomes among cancer survivors are still under-researched. A comprehensive understanding of these challenges and their associations could help to manage these complications early in the treatment process, treatment strategies, and survival outcomes. The evidence could also help develop tailored interventions for improving nutritional and psychological status, health-related quality of life, and long-term outcomes of cancer survivors.

This Research Topic aims to gather updated evidence on the interrelationships between nutritional biomarkers, mental status, and prognosis among cancer survivors. Five peer-reviewed papers were collected, covering the above-mentioned topics. This editorial aims to synthesize the findings of these papers, and further discuss their implications for clinical practice and future research directions.

Lung, female breast, colorectum, and prostate cancers are the top four most frequently diagnosed cancers in 2022, with lung cancer also being the leading cause of cancer mortality worldwide (8). The consumption of ultra-processed food (UPF) is rapidly increasing globally and is significantly associated with an increased risk of various health conditions, including cancers such as colorectal cancer, breast cancer, and ovarian cancer (9–11). Pu et al. further investigated the association between pre-diagnosis UPF consumption and the prognosis of patients with colorectal, lung, prostate, or breast cancer. They found that high UPF consumption before cancer diagnosis was significantly associated with an elevated risk of all-cause mortality in patients with lung or prostate cancer, with a non-linear dose-response trend, and also increased the risk of cancer-specific mortality in patients with early colorectal cancer or prostate cancer with low body mass index. The results indicate that reducing UPF consumption before cancer diagnosis may improve the overall or cancer-specific survival of these patients. Zhao et al. conducted an ambispective cohort study to investigate the impact of dietary components, specifically phytosterols, on ovarian cancer survival, revealing a potential protective effect of pre-diagnosis higher dietary intake of campesterol, stigmasterol, and β-sitosterol on better overall survival. The results underscore the importance of dietary components in cancer care. These papers contribute to the growing body of evidence linking better dietary quality (e.g., Mediterranean diet) to improved cancer survival (12), highlighting the importance of dietary recommendations in cancer care beyond conventional treatments.

Blood biomarkers are often used to evaluate the nutritional status of cancer patients. For example, serum triglyceride (TG), associated with diet, is treated as a nutritional marker. Previous studies indicate that a high TG level has different effects on tumorigenesis and prognosis in cancer patients. A high TG level is a risk factor for cancer development involving cell proliferation and tumor growth (13, 14), while some studies report a positive association between preoperative lower TG level and a worse prognosis for cancer patients (15). Lyu et al. presented a novel perspective, identifying post-treatment serum TG levels as a significant prognostic biomarker for body fat mass and overall survival in patients with esophageal squamous cell cancer. A post-treatment TG level of ≥1.47 mmol/L is an independent predictor for better overall survival. The findings indicate that lipid metabolism may play a crucial role in cancer outcomes, which encourages further investigation into the use of serum TG as a potential therapeutic target and prognostic indicator in cancer care. The study of Li et al. introduces a novel nutritional index set by combining several blood markers (e.g., albumin, total protein, total cholesterol, electrolytes, and granulocytes) to predict lung cancer risk and treatment outcomes.

In the longitudinal study of Wang et al., the authors highlighted the high incidence of nutritional risk, defined as a total score ≥3 points measured by the Nutritional Risk Screening 2002 scale, among perioperative oral cancer patients, with the trajectory of nutritional risk and score fluctuating from admission day to 1-month post-discharge. The highest incidence of nutritional risk and score were observed at 7 days post-surgery. They also reported a positive correlation between nutritional risk and mental problems (i.e., depression and anxiety). By identifying malnutrition as a significant challenge, the findings emphasize the importance of nutritional monitoring and management, particularly in postoperative patients or those with low educational level, advanced-stage cancer, flap repair, tracheotomy, or low body mass index, to enhance recovery and survival outcomes.

In summary, the results from the above-mentioned studies emphasize the multifaceted role of nutrition in cancer survivorship, underscoring the need to consider nutrition as a critical component of cancer management. These findings highlight the importance of understanding how dietary habits and nutritional biomarkers impact cancer treatment and prognosis outcomes. Personalized dietary assessments and interventions, covering the full journey of cancer survivors, are highly warranted. However, it is regrettable that no studies covering the field of mental health and the prognosis of cancer survivors were included in this Research Topic. More studies on the mental health aspect of cancer survivors are highly warranted, including the prevalence, longitudinal trajectory during and after the treatment, their influence on prognosis, and health-related quality of life. This aligns with the growing recognition in the psycho-oncology field, which addresses the psychological and behavioral aspects of cancer care. Additionally, future research should continue to explore dietary interventions and biomarkers that could improve patient outcomes while considering the psychological and social aspects of cancer survivors. The integration of these insights into clinical practice has the potential to transform cancer care by offering patients a more holistic and targeted approach to treatment and recovery.

## Author contributions

J-BL: Conceptualization, Data curation, Formal analysis, Funding acquisition, Investigation, Methodology, Project administration, Resources, Software, Supervision, Validation, Visualization, Writing – original draft, Writing – review & editing.
